# How International Internships Enrich a Medical Career

**DOI:** 10.21470/1678-9741-2018-0142

**Published:** 2018

**Authors:** Beatriz J. S. Branco, Camila R. Ribeiro

**Affiliations:** 1 Faculdade de Medicina da Universidade de Mogi das Cruzes, Mogi das Cruzes, SP, Brazil.; 2 Faculdade de Medicina do Centro Universitário de Brasília (UniCEUB), Brasília, DF, Brazil.

"I am the master of my fate,

I am the captain of my soul."

William Ernest Henley

As medical students, we always want to thrive and grow beyond the walls of our universities and hospitals. Having that will in our hearts, we started our journey in our own cities, Mogi das Cruzes-SP, and Brasilia-DF, both in Brazil. Not knowing each other, we heard about The William J. Harrington Medical Training Program for Latin America and the Caribbean at the University of Miami Miller School of Medicine. In view of the professional and intellectual relevance that an exchange can provide, in addition to the opportunity for personal and cultural growth, the choice to participate in this program has presented itself as a major challenge: totally new and unknown context. However, it would undoubtedly be a great and exciting experience.

The program offers a wide range of opportunities. Since its inception, more than 3,500 international physicians and medical students have participated in observerships^[1]^. It accepts hundreds of medical students per year into rotations in every medical specialty, its subspecialties and in other areas of Medicine. It allows students to participate in morning rounds, clinics, watch surgeries, prepare and attend case presentations, lectures and seminars. We had the opportunity to see how different Medicine could be in different countries and how the advances in Medicine take long to arrive at our country. Thus, one study on medical education shows that international exchanges allows students to behold different models of doctor-patient relationship and interacting with different professionals could improve their social and intercultural skills^[2]^.

**Fig. 1:**
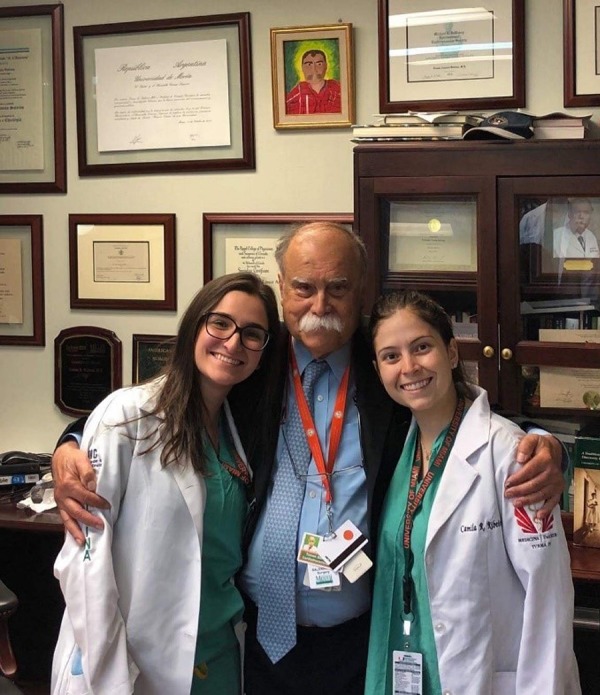
Tomas Salerno, MD (middle), and the medical students Beatriz Branco and Camila Ribeiro (left and right, respectively).

We both had the privilege to rotate with the Cardiothoracic Surgery team at the Jackson Memorial Hospital, which is exceptionally led by Tomas Salerno, MD. Salerno is a professor of surgery, Vice Chairman for Faculty Development and Mentoring in the Department of Surgery, and Chairman of the University of Miami Faculty Senate. Besides his great ability to teach, Dr. Salerno showed himself as a brilliant and passionate physician who absolutely loves to operate and treat every patient as his own family. Besides that, he turned out to be an example of humanity and dedication: in his seventies, he still goes to the hospital on weekends (Figure 1). It was our honor to participate in his surgeries and learn how Medicine does not only consist of treating diseases. Medicine involves the patients, families, nurses and all the healthcare professionals.

One of Dr. Salerno's teachings lie on how frustrating it can be to a physician dealing with a noncompliant patient. When a patient fails to follow clear instructions and gets into a worse state than before, it is hard to deal with frustration and which part of the guilt could be truly attributed to the physician^[3]^. Surgeons, specially, have a difficult time trying to decide who will benefit from the surgery and who will benefit from no operation at all. The Cardiothoracic Surgery department at Jackson does not stop, there were different cases to participate every day, such as: valves replacement, pericardial window, heart transplants, and, daily, coronary artery bypass graft. Oftentimes, Dr. Salerno would perform a coronary bypass without the assistance of another surgeon in the room, using the off-pump technique.

Coincidentally, Professor Salerno is Brazilian, from the state of Minas Gerais, and his name is internationally prestigious in cardiac surgery. The Brazilian cardiac surgery is known to be the house of important advances in surgery techniques, such as the beating heart revascularization technique, the use of double internal mammary grafting, and the current surgical treatment of heart failure. Thus, in 1958, Brazilian doctors developed the extracorporeal circulation equipment and ten years later, in 1968, the first heart transplant in Brazil was performed by doctor Euryclides de Jesus Zerbini, only some months after Christiaan Barnard performed the world's first heart transplant in South Africa^[4]^.

While rotating in the service, the cardiothoracic surgery team had five attendings, three fellows and two interns - all men. Although we saw women in general surgery, men were clearly the majority. From this experience abroad and from the experience in Brazil, it is evident that women are, still, under-represented in academic medicine. Two of the four consistent evidences found in one review study that aimed to discuss the reasons women had to choose or deny spots in academic medicine were that women lack appropriate mentors and role models and, sadly, they still find trouble with gender discrimination and bias^[5]^.

Furthermore, the construction of a "parallel curriculum" is proved to be enriching to shape the future doctor and for the personal development of medical students. In Brazil, studies at six different medical schools showed that, by the third year of medical school, students were, somehow, already connected to extracurricular activities, such as academic leagues, internships and monitoring programs. The students who were evaluated tended to look for extracurricular activities in order to, mostly, improve the formal curriculum of medical schools, to refine their medical abilities and to have a competitive curriculum to apply for medical residencies^[6]^. One of students' major concerns when they get to the sixth year of medical school - the last, in Brazil - is to study for the residency exams. Each service has their own exam and the competition is high to get a spot at a well-known and resourceful hospital. A newspaper has just published that in 2018, 40% of the residency spots on the country were vacant, because of, mostly, lack of preceptors and quality programs^[7]^. In addition, the proficiency in a second language, mainly English, is proved to be expected from any decent resume, considering that medical advances and new publications are available first-hand in other languages.

Besides the fact that an international internship would enrich our resumes, we also get to test our capacity of working in groups and our fluency in English to use it in favor of our personal inclinations to humanitarian missions. The attention given to missions in controlling infectious diseases has shifted over the past decades to aid against non-transmissible diseases, such as cardiovascular disease, which remains the leading cause of death worldwide. Therefore, cardiac surgery and cardiology missions' formats can be varied, from providing preventive clinical care to managing direct surgical treatment^[8]^.

Considering the observership in its entirety, we could only say that there was no better way to improve our medical capacity and expand our cultural boundaries if not challenging ourselves to dwell between the walls of an unknown hospital, city and people.

**Table:** 

Authors' roles & responsibilities	
BJSB	Substantial contributions to the conception or design of the work; final approval of the version to be published
CRR	Substantial contributions to the conception or design of the work; final approval of the version to be published
